# Clinical outcome measures in dementia with Lewy bodies trials: critique and recommendations

**DOI:** 10.1186/s40035-022-00299-w

**Published:** 2022-05-02

**Authors:** Federico Rodriguez-Porcel, Kathryn A. Wyman-Chick, Carla Abdelnour Ruiz, Jon B. Toledo, Daniel Ferreira, Prabitha Urwyler, Rimona S. Weil, Joseph Kane, Andrea Pilotto, Arvid Rongve, Bradley Boeve, John-Paul Taylor, Ian McKeith, Dag Aarsland, Simon J. G. Lewis, the Lewy Body Dementias Clinical Trials Workgroup from the Lewy Body Dementias Professional Interest Area - Alzheimer’s Association International Society to Advance Alzheimer’s Research and Treatment (ISTAART)+

**Affiliations:** 1 Department of Neurology, Medical University of South Carolina, 208b Rutledge Av., Charleston, SC 29403 USA; 2 Department of Neurology, Center for Memory and Aging, HealthPartners, Saint Paul, MN USA; 3 Autonomous University of Barcelona, Barcelona, Spain; 4 Fixel Institute for Neurological Diseases, University of Florida, Gainesville, FL USA; 5 Division of Clinical Geriatrics, Department of Neurobiology, Care Sciences, and Society, Center for Alzheimer’s Research, Karolinska Institutet, Stockholm, Sweden; 6 Department of Radiology, Mayo Clinic, Rochester, MN USA; 7 ARTORG Center for Biomedical Engineering Research, University of Bern, Bern, Switzerland; 8 Dementia Research Centre, University College London, London, UK; 9 Centre for Public Health, Queen’s University, Belfast, UK; 10 Neurology Unit, Department of Clinical and Experimental Sciences, University of Brescia, Brescia, Italy; 11 Department of Research and Innovation, Helse Fonna, Haugesund Hospital, Haugesund, Norway; 12 Institute of Clinical Medicine (K1), The University of Bergen, Bergen, Norway; 13 Department of Neurology, Center for Sleep Medicine, Mayo Clinic, Rochester, MN USA; 14 Translational and Clinical Research Institute, Newcastle University, Newcastle upon Tyne, UK; 15 Department of Old Age Psychiatry Institute of Psychiatry Psychology and Neuroscience, King’s College London, London, UK; 16 ForeFront Parkinson’s Disease Research Clinic, Brain and Mind Centre, School of Medical Sciences, University of Sydney, 100 Mallett Street, Camperdown, NSW 2050 Australia

**Keywords:** Dementia with Lewy bodies, Clinical trials, Outcomes, Measurement properties

## Abstract

The selection of appropriate outcome measures is fundamental to the design of any successful clinical trial. Although dementia with Lewy bodies (DLB) is one of the most common neurodegenerative conditions, assessment of therapeutic benefit in clinical trials often relies on tools developed for other conditions, such as Alzheimer’s or Parkinson’s disease. These may not be sufficiently valid or sensitive to treatment changes in DLB, decreasing their utility. In this review, we discuss the limitations and strengths of selected available tools used to measure DLB-associated outcomes in clinical trials and highlight the potential roles for more specific objective measures. We emphasize that the existing outcome measures require validation in the DLB population and that DLB-specific outcomes need to be developed. Finally, we highlight how the selection of outcome measures may vary between symptomatic and disease-modifying therapy trials.

**Supplementary Information:**

The online version contains supplementary material available at 10.1186/s40035-022-00299-w.

## Introduction

Dementia with Lewy bodies (DLB) is the second most common type of neurodegenerative dementia after Alzheimer's disease (AD) [1]. Compared to AD, DLB is associated with a poorer prognosis, higher healthcare costs and caregiver burden, and greater impact on quality of life [2]. Currently, symptomatic therapies are limited in DLB and there are no disease-modifying therapies (DMTs). However, disease-modifying approaches are being strongly pursued in other neurodegenerative conditions, for example, amyloid beta-directed monoclonal antibodies for AD and genetic therapies targeting glucocerebrosidase mutations in Parkinson’s disease (PD) [3, 4]. Therefore, establishing robust outcome measures is crucial to permit the evaluation of symptomatic treatments and DMTs in DLB. To date, no specific DLB outcome measures have been validated for use in clinical trials, which have typically relied on scales developed for AD and PD [5] (Table 1). Previously, regulatory authorities have emphasized the need for outcome measures that address functional, cognitive, and global domains in AD, but guidance is lacking in DLB [6–8].

**Table 1:** Outcomes used in DLB clinical trials

Domain	Assessed in clinical trials: *n* (%)	Primary outcome: *n* (%)	Most common method used (*n*)
Functional	22 (52)	5 (11)	ZBI (7)
Cognitive	39 (92)	17 (40)	MMSE (33)
Visual hallucinations	35 (83)	21 (50)	NPI (22)
Fluctuations	9 (21)	1 (2)	Mayo FSI (4)
Motor	28 (66)	10 (23)	UPDRS-III (23)
RBD	1(2)	1 (2)	RBDSS scale (1)

*RBD* REM sleep behavior disorder, *ZBI* Zarit Burden Interview, *MMSE* Mini-Mental State Examination, *NPI* Neuropsychiatric Inventory, *Mayo FS* Mayo Fluctuation Scale, *UPDRS-III* Unified Parkinson’s Disease Rating Scale Part III, *RBDSS* RBD Severity Scale

Randomized clinical trials, the highest level of evidence to guide treatment, are scarce among DLB cohorts, and most of the experience comes from case studies or open-label observations without control groups. There are multiple challenges in conducting a clinical trial in DLB, including delayed and inaccurate diagnosis, significant clinical heterogeneity, and the use of concomitant medications [9]. However, one of the greatest challenges in conducting robust DLB clinical trials is the selection of appropriate outcome measures. While choice of intervention, population, and trial design are all intuitively important, without the selection of an appropriate outcome and a rigorous method to measure it, any trial could be unrevealing or misleading [10]. The appropriate outcome measures will not only affect the accuracy and applicability of the trial, but also have implications for initial sample size estimations [10].

Outcome measures typically rely on clinical assessments rather than objective measures and whilst the severity of the measure may be implied by its score, this has typically not been validated in DLB. Additionally, any outcome measure needs to consider the type of intervention being evaluated (symptomatic or DMT), as well as the disease severity of the population being studied. For example, whilst symptomatic therapies will likely focus on measurement of the specific domain being targeted, DMTs may focus on the time to the development of clinical milestones. Moreover, the progression of the disease may affect clinical domains differentially, wherein changes in one feature may not be a suitable marker of overall progression [11]. There is also an unresolved issue around what might constitute a meaningful clinical change or minimal clinically important difference (MCID) [12]. Many outcome measures in DLB are also likely to be impacted by poor patient recall, and reliance on caregivers may introduce recall bias and failure to report clinical features that may be difficult to directly observe (e.g., hallucinations). Finally, disease-specific factors can also present their own set of unique challenges in DLB trials, such as where one symptom might impact another (e.g. bradykinesia affecting cognitive response time tests); assessment sessions that coincide with cognitive fluctuations; and, the potential effects of concomitant medications (e.g. increased parkinsonism from antipsychotics or anti-depressants).

This paper represents the first in a series by the Clinical Trials Workgroup of the Lewy Body Dementias Professional Interest Area—Alzheimer's Association International Society to Advance Alzheimer's Research and Treatment (ISTAART). In this paper, we will focus on the tools for measuring clinical outcomes (e.g., rating scales, cognitive batteries), whilst biomarkers, such as imaging and cerebrospinal fluid biomarkers, will be discussed elsewhere in our series. In this article, we discuss the outcome measures previously used in DLB clinical trials and specifically evaluate what might be considered necessary in future work to identify novel symptomatic and disease-modifying therapies in DLB that would meet the requirements of the regulatory agencies. In addition to cognitive and functional outcomes, we will also focus on measuring the core features of DLB, which include cognitive fluctuations, visual hallucinations, parkinsonism, and rapid eye movement sleep behavior disorder (RBD) [13]. A discussion regarding the outcome measures to address other aspects of the disease (e.g., autonomic function or mood, fatigue, specific cognitive domains) is beyond the scope of our review but will be addressed in the future by our working group.

### Assessment of clinical outcomes: measurement properties

Clinical outcomes are classified either as the occurrence of an event or milestone, or changes in clinical measures (Table 2). Such outcome measures may be obtained either by direct observation and quantification or by reports from patients and/or their caregivers [10]. Measurements can range in scope, from global functionality to a specific aspect of the condition.

**Table 2:** Types of outcome measures

Outcome measure	Example	Advantage	Disadvantage
Direct/objective	Falls/death	Easy to measure	May not capture the symptom severity or impact on daily life
Performance outcome	Neuropsychological evaluation	Objective measure	Only evaluating one point in time—not a reflection of the current status
			Artificial environment
Clinician reported outcome	Clinical scales	Clinical relevance	Depends on expertise of administrator
Patient-reported outcome	Quality of life questionnaires	Centered on the patient	Impaired cognition may affect reliability of responses
Observer-reported outcome	Activities of daily living questionnaires	Provides a better assessment of daily function	Subject to bias or caregiver’s cognitive ability

When selecting a clinical outcome measure, there are three major considerations: i) measurement properties, ii) interpretability of the results, and iii) feasibility. The measurement properties reflect the tool’s ability to measure a specific variable in the group of interest (validity), the random error associated with the outcome measure (reliability), and its sensitivity to detect change (responsiveness) [14]. Assessments of these domains are crucial for the validation of outcome measures and can help to identify the appropriate conditions under which each measure is used (Table 3). Interpretability refers to the degree to which one can assign qualitative meaning to the outcome measure’s quantitative scores or change, including the distribution of scores within subgroups to assess for any floor or ceiling effects [14]. One of the most important concepts within interpretability is that of the MCID, defined as the smallest amount that an outcome must change to be meaningful to patients [12]. It is recognized that there are different methods to determine the MCID (e.g., anchor-based and distribution-based), but these are beyond the scope of this paper [12]. Establishing what might constitute an MCID is fundamental to designing studies with sufficient statistical power to detect an effect [15]. Finally, feasibility assesses the practical aspects of the outcome measure, such as the burden to the patient, caregiver, and administrator. This is particularly relevant for dementia research, where subjects may not be able to tolerate prolonged and intense periods of evaluation.

**Table 3:** Measurement properties of outcome measures

Domain	Measurement property	Definition	Methods of assessment
Reliability		The extent to which an OMI is free from random error	
	Reproducibility	The proportion of the total variance in the measurement due to true differences between patients. Includes Inter-rater rand test–retest reliability	Kappa coefficient
			Interclass correlation coefficient
	Internal consistency	The degree of interrelatedness among the items	Cronbach’s alpha
			Item-total correlations
			Homogeneity coefficient
	Measurement error	The systematic and random error of a patient’s score that is not attributed to true changes in the construct to be measured	Standard error of measurement
			Bland–Altman method
Validity		The extent to which an OMI the construct that it is supposed to measure	
	Content validity	The degree to which the OMI covers the important parts of the construct to be measured	Lynn’s Content Validity Assessment from an expert panel
	Construct validity	The degree to which the scores of an OMI are consistent with the expected scores, based on the existing knowledge of the construct. Includes structural, hypothesis-testing, and cross-cultural validity	Structural validity: Factor analysis
			Hypothesis-testing: Convergent, divergent, and internal validity compared to another validated measure
			Cross-cultural: measurement invariance in different populations
	Criterion validity	The degree to which the scores of an OMI are an adequate reflection of the gold standard (if available)	Criterion-outcome measure agreement or correlation coefficient
Responsiveness	Responsiveness	The ability to detect change over time in the construct to be measured	Effect size
			Standardized response mean
			Responsiveness statistic

Initiatives such as the Core Outcome Measures in Effectiveness Trials (COMET) and the COnsensus-based Standards for the selection of health Measurement Instruments (COSMIN) provide practical guidelines on how to select the appropriate outcome measurement instrument [16–18].

## Methods

In this paper, we focused on clinical outcome measures used in DLB trials. We conducted a literature review of clinical trials in patients with DLB. Using PubMed, we searched for pharmacological or neurostimulation trials in DLB patients published between January 1975 and March 2021. We used a combination of the following search terms: “Dementia with Lewy bodies”; “Lewy body dementia”; “Lewy body”; “clinical trial”, “therapy”; “management”; and “treatment”. A total of 2079 non-duplicate citations were identified. One reviewer (FRP) then screened the abstracts and full-texts according to the following inclusion criteria: (1) Prospective studies evaluating pharmacological modulation or neuromodulation and (2) studies of participants with DLB alone or in combination with AD, PD, or PD dementia. Retrospective studies, extension studies, and meta-analysis were excluded. A total of 42 trials were included. The outcome measures utilized in the clinical trials were then classified into 7 domains: (1) neuropsychological; (2) neuropsychiatric; (3) motor; (4) fluctuations; (5) autonomic; (6) sleep; and (7) activities of daily living and quality of life. In addition, we included other instruments that the authors found promising or worthy of discussion, even not yet used in DLB clinical trials.

A separate search was conducted to evaluate the measurement properties of the clinical outcome measures. For all outcome measures, we used PubMed to perform an additional search of empirical studies assessing their validity and reliability, using the search terms: “clinimetric”; “clinical significance”; “clinically meaningful change”; “validity”; “validation”; “responsiveness”; “reliability”; and “sensitivity to change”. The quality of the studies was evaluated using the COSMIN checklist for assessing the methodological quality of studies on measurement properties of health status measurement instruments and the guideline to select outcome measurement instruments for outcomes [16, 19]. Based on these guidelines, if the outcome measures passed the acceptable cutoffs in one or more high-quality studies, they were determined as “good/adequate”. If there was conflicting evidence, or if there was evidence showing the outcome measures did not pass acceptable cutoffs, they were considered “questionable/mediocre”. If an MCID was published for neurodegenerative dementia, this information was included. Although no MCID values have been published for DLB, we included those published for other conditions, such as AD and PD, considering them to be a helpful guide.

### Functional, quality of life, caregiver burden, and global impression outcomes

The term functional outcome is used as the measure of how a patient can successfully perform the meaningful tasks and roles required as part of typical everyday life [10]. Such real-world measures are important efficacy endpoints for clinical trials in DLB and are usually required by regulatory authorities. In addition, quality of life and caregiver burden are also relevant and warrant measurement. Finally, the measurement of multiple aspects of function can answer the most important question of a clinical trial, i.e., whether the intervention produces a meaningful improvement in the patient’s condition. Therefore, the use of a clinical impression measurement is regarded as a useful and necessary outcome, particularly in symptomatic trials.

The Alzheimer’s Disease Cooperative Study-Activities of Daily Living Scale (ADCS-ADL) is one of the most widely used functional scales in clinical trials. It evaluates the ability of the patient to perform basic and instrumental activities of daily living (iADLs) and is administered only to the caregiver. The ADCS-ADL has previously been used in several trials for other conditions, such as AD and PD dementia (PDD) [20]. While its measurement properties have not been established for DLB, the ADCS-ADL is a valid and reliable measurement, sensitive to clinical progression in AD [20, 21]. Although a change of 2 points has been suggested to be clinically meaningful in AD, this has not been evaluated formally [20, 21]. In PDD, the ADCS-ADL has demonstrated responsiveness in detecting treatment effects, which makes it a promising scale for DLB trials [22]. The Disability Assessment for Dementia (DAD) scale is a subscale of the Clinician’s Interview-Based Impression of Change scale [23]. While the DAD shows validity and reliability in AD trials, its sensitivity to AD clinical progression or responsiveness to treatment has not been consistent [24–26]. Part II of the Movement Disorders Society Unified Parkinson's Disease Rating Scale (MDS-UPDRS) assesses activities of daily living but is not specific to dementia and is not responsive to detecting treatment effects in PD, limiting its validity [22, 27]. The Schwab and England ADL scale is widely used in PD, but the extent to which it is valid in dementia trials has also not been evaluated [28]. Technology-based objective measures (TOMs) offer an alternative to questionnaires [29]. Options range from motion sensors to smart home technology, which consists of a combination of sensors and connected devices that monitor (and control if needed) the use of appliances at home [30]. These systems can monitor several iADLs at home, such as the ability to perform online banking or the time spent watching television or sleeping [31]. While these technologies are already available in the community, the translation of the data collected to meaningful trial outcomes remains to be determined, particularly in DLB.

Other important clinical aspects include quality of life measures and caregiver burden scales. Considering that the goal of disease treatment is the improvement in a patient’s quality of life, these measures should be incorporated within clinical trials as secondary outcomes. Quality of life measures have only rarely been used in DLB trials with the Quality of Life in Alzheimer’s Disease Scale (QOL-AD) being used once to date [32]. In AD and PDD, the QOL-AD shows good validity and responsiveness to treatment effects, but this has not been validated in DLB [22]. Alternatives include the 39-item Parkinson’s Disease Questionnaire (PDQ-39), which has good validity and reliability for PD and for which the MCIDs for the different domains included in the scale (e.g., mobility, ADLs) have been determined [33]. The PDQ-39 has been used in one PDD trial, but it may be insensitive to clinical progression [22, 34]. The impact on caregiver burden might also provide meaningful information regarding the impact of an intervention. The Zarit Burden Interview (ZBI) was developed to measure caregiver burden related to behavioral and functional impairment in dementia patients [35]. Although it has been shown to be responsive to intervention, the ZBI has demonstrated mixed results in attempts to correlate its findings with other clinical measures, such as cognition and sleep [22, 36]. The Relative Stress Scale is an alternative to the ZBI and has been used in AD and PD trials as well as in observational studies in DLB [37–39].

Global impression scales can be useful in capturing treatment effects that may go unmeasured by other scales. The Clinician’s Interview-Based Impression of Change scale plus combines clinician and caregiver input. Measures of clinically meaningful change are intrinsic to the scale, which have been shown to be responsive to treatment effects in AD and PDD [22, 23]. The Clinical Global Impression Scale (CGI) consists of scales evaluating three aspects: severity, improvement, and efficacy. Similar to the Clinician’s Interview-Based Impression of Change Scale (CIBIC+) , the scale measures meaningful clinical change per se and has demonstrated responsiveness to treatment effects in AD trials [22]. In addition, the CGI has been used in PDD and DLB trials, including one trial that showed benefit from memantine [40]. Both the CIBIC and the CGI exhibit a good correlation with the Clinical Dementia Rating-Sum of Boxes in AD [41]. The Alzheimer’s Disease Cooperative Study–Clinician’s Global Impression of Change (ADCS-CGIC) is one of the most commonly used scales in dementia trials [42]. While responsiveness to treatment effects has been noted in PDD and DLB trials, the lack of correlation with the Functional Assessment Staging Scale warrants the use of the ADCS-CGIC as a complement to other ADL scales (Table 4) [22, 23, 43].

**Table 4:** Selected functional, quality of life, caregiver burden and global impression outcomes

Outcome	Type of measure	Rater	Reliability	Responsiveness	MCID	Used in DLB trials
ADCS-ADL	ADLs	Informant	+	+	2 points	Yes [44–46]
DAD	ADLs	Informant	+	+	NE	Yes [40, 47]
UPDRS-II	ADLs	Informant Subject	NE	NE	NE	Yes [48, 49]
SEADL	ADLS	Informant	NE	NE	NE	No
QOL-AD	QoL	Subject	+	+	NE	Yes [32]
PDQ-39	QoL	Subject	+/−	NE	NE	No
ZBI	CB	Informant	+/−	+	13 points	Yes [43, 45, 47, 50–53]
RSS	CB	Informant	+	NE	NE	No
CIBIC+	IoC	Clinician/Informant	+/−	+	NE	Yes [43, 54–58]
CGI	IoC	Clinician	+/−	+	NE	Yes [54, 59, 60]
ADCS-CGIC	IoC	Clinician	+/−	+	1 point	Yes [44, 45, 61, 62]

+, good/adequate; +/−, acceptable; −, performance is questionable/mediocre. *ADLs* activities of daily living, *CB* caregiver burden, *IoC* impression of change, *MCID* minimal clinically important difference, *NE* not evaluated, *QoL* quality of life, *ADCS-ADL* Alzheimer’s Disease Cooperative Study—Activities of Daily Living Scale, *DAD* Disability Assessment for Dementia, *UPDRS-II* Unified Parkinson’s Disease Rating Scale Part II, *SEADL* Schwab and England Activities of Daily Living, *QOL-AD* Quality of Life in Alzheimer’s Disaese Scale, *PDQ-39* 39-Item Parkinson’s Disease Questionnaire, *ZBI* Zarit Burden Interview, *RSS* Relative Stress Scale, *CIBIC*+ Clinician’s Interview-Based Impression of Change Scale, *CGI* Clinical Global Impression Scale, *ADCS-CGI* Alzheimer’s Disease Cooperative Study–Clinician’s Global Impression of Change

Currently, we suggest that a combination of an activities of daily living scale and a clinical impression of change would be necessary to determine the clinical effectiveness of an intervention in DLB. Furthermore, we propose adding an assessment of quality of life and caregiver burden as secondary aims to provide meaningful additional information on the impact of any therapy being evaluated. Future consideration could be given to collecting objective digital outcomes from wearable devices [29]. We recommend that the development of specific DLB functional and global assessment instruments also consider the importance of differentiating the degree to which cognitive, behavioral (including sleep), autonomic, and motor symptoms all contribute to the functional impairment experienced by DLB patients.

### Cognitive outcomes

Cognitive impairment is an essential criterion for the diagnosis of DLB and the primary target outcome for most clinical trials conducted to date. The neuropsychological profile in DLB is typically characterized by impairments in attention, executive functioning, and visuospatial abilities in addition to memory [13]. However, heterogeneity in cognitive test performances is common, and impairment in language can also be present, particularly at later clinical stages of the disease [63, 64]. While the deficits in different cognitive domains are attributed to the pathological processes and distribution of neuropathology in DLB, other pathologies (e.g., cerebrovascular disease) and pathophysiologic mechanisms (e.g., cognitive fluctuations), may impact cognitive performance differently. This may affect the potential response to treatment, in both symptomatic and disease-modifying therapy trials [65, 66].

The inclusion of a global cognitive measure is recommended for all DLB clinical trials. The Mini-Mental State Examination (MMSE) has been utilized as a global measure in numerous DLB studies [67, 68]. Although it has proven to be reliable, the validity of the MMSE in DLB has been questioned, mainly due to its limited testing of executive function [69]. Furthermore, the MMSE has modest sensitivity to cognitive deficits in patients with autopsy-confirmed DLB [70]. In addition, its floor effects in patients with severe dementia, its ceiling effects in patients with mild cognitive impairment, and its lack of responsiveness to small changes noted early in PDD, limit its use as a cognitive endpoint for clinical trials in DLB [71–73]. However, it has been useful in showing the benefit of donepezil in DLB and was identified as a potential marker for change over just a 6-month interval in a recent natural history study, suggesting possible utility in future DMTs [43, 74]. The Montreal Cognitive Assessment (MoCA) is a reliable assessment that includes items that assess attention (e.g., trail making test part B) and executive functions like working memory (e.g., digit span backward), making it a more valid instrument for DLB trials and accepted as a measure of change in regulatory trials. Previous studies have shown that MoCA is certainly more sensitive than the MMSE for the detection of cognitive impairment in DLB, particularly early-on in the disease [72, 75, 76]. However, its ability to detect subtle cognitive changes remains to be determined. The Alzheimer’s Disease Assessment Scale-Cognitive (ADAS-Cog) has also been utilized in several clinical trials of DLB [68, 77]. The addition of tests of executive functioning and attention to the ADAS-Cog can increase the ability to detect cognitive decline in non-AD dementias, although this measure may be insensitive in the earliest stages [78, 79]. The ADAS-Cog has been shown to be responsive to change in PDD and AD and one group analysis has defined the MCID as 4 points in AD [80]. The Mattis Dementia Rating Scale (MDRS) has also been used in multiple trials and has been validated for PDD but not DLB. The MDRS seems to be superior in the assessment of cognition when compared to the MMSE and has also shown good responsiveness in trials assessing PDD [22, 81] (Table 5).

**Table 5:** Selected general cognition outcomes

Outcome	Detection	Discrimination	Reliability	Responsiveness	MCID	Used in DLB trials
MMSE	+*	+*	+	+/−	NE	Yes [40, 43, 44, 47, 52, 54–58, 60–62, 82–101]
MoCA	+*	+*	+	+/−	NE	Yes [46]
ADAS-CoG	NE	NE	+	+	4 points	Yes [44–46, 48, 58, 62]
MDRS	+	NE	+	+	NE	Yes [32, 59, 83]
RBANS	+	NE	+	+	NE	No
COGDRAS	NE	NE	+	+	NE	Yes [40, 44, 55, 57, 88]

*MCID* minimal clinically important difference, *NE* Not evaluated, *MMSE* Mini Mental State Examination, *MoCA* Montreal Cognitive Assessment, *ADAS-CoG* Alzheimer’s Disease Assessment Scale-Cognitive, *MDRS* Mattis Dementia Rating Scale, *RBANS* Repeatable Battery for the Assessment of Neuropsychological Status, *COGDRAS* Cognitive Drug Research Computerized Assessment System

+, good/adequate; +/−, acceptable; −, performance is questionable/mediocre

*Evaluated in DLB population

In addition to measures of global cognitive performance, the use of more specific neuropsychological tests in clinical trials has several strengths, including established normative data for standardization of performances, and availability of published literature regarding psychometric properties such as reliability, construct validity, and practice effects in dementia (although not in DLB). Neuropsychological tests are also more sensitive to cognitive impairment than measures of global cognition and can more effectively assess individual domains [102] (Additional file 1: Table S1). However, neuropsychological batteries may be too lengthy or cumbersome to routinely administer. Brief batteries utilizing the same normative data source, such as the Repeatable Battery for the Assessment of Neuropsychological Status, have not been utilized in previous DLB clinical studies but may be a consideration for future trials.

Computerized cognitive assessments could also have several strengths, including standardized administration and automated scoring. These can include a stand-alone computerized test within a larger battery or a fully automated battery. Many computerized assessment measures can be administered remotely with generally similar results as in-clinic evaluations and can provide increased sensitivity to change compared to pen-and-paper tests, reducing the required sample sizes, which would be helpful in reducing the difficulty to recruit populations like DLB [103]. Computerized tests assessing reaction time and vigilance may be more sensitive in detection of cognitive fluctuations than traditional paper-and-pencil measures and may be better suited to capture cognitive fluctuations over short timescales, although they may not capture longer more profound fluctuations that occur over hours or days. In addition, computerized testing may allow dynamic adaptation of the degree of difficulty of the testing, which would reduce floor and ceiling effects. Several computerized batteries are sensitive enough to detect a cognitive change in clinical trials for other neurodegenerative diseases, and the Cognitive Drug Research Computerized Assessment System has been used in multiple DLB trials [44, 55, 57, 104–110]. Whilst such computerized cognitive assessments appear promising, there is a clear need for their further development and validation in DLB [104, 111, 112].

We recommend that, at a minimum, DLB clinical trials should include a measure of global cognition, acknowledging that whilst quick to administer, they are not always sensitive in individuals with high levels of education or those in the early stages of the disease [72, 113–117]. The use of a global composite cognitive score is promising but may fail to identify the degree of impairment within a single cognitive domain [118, 119]. The selection of any specific cognitive outcome measure may also depend on the therapeutic target. Symptomatic therapies addressing specific domains, such as attention or executive function, should consider specific testing, as an outcome, in addition to a general cognitive measure. Furthermore, cognitive outcomes in DMTs will depend on the stage of the condition being evaluated. Whilst measures of attention, executive and visuospatial function seem to be regarded as being better predictors for the transition from MCI-LB to dementia in DLB, measurements of language and memory function appear to be more sensitive to decline across dementia stages, although further validation is needed [11, 120, 121].

### Cognitive fluctuations

Fluctuating cognition is a characteristic feature of DLB and forms part of the core diagnostic criteria [13]. Cognitive fluctuations present as cognitive changes secondary to impairments in attention or somnolence due to variations in alertness [122]. The assessment of cognitive fluctuations in clinical trials may serve one of two purposes: (i) as a primary symptom or disease treatment target or (ii) assessing a potential confounder in the performance of other domains, such as cognition. The choice of assessment will vary depending on the goal of the measurement.

The Clinician Assessment of Fluctuation (CAF) 4-item scale is a short tool that needs to be administered by an experienced clinician. While the CAF has been correlated with neuropsychological and electrophysiological measures, it assumes that severity is dependent on frequency and duration, which can affect its validity and use in clinical trials [123]. The CAF has shown nearly perfect interrater reliability in severe cognitive fluctuations, while the reliability to establish the presence/absence of fluctuations is only fair [124]. The Mayo Fluctuations Scale (19-item questionnaire) is a comprehensive questionnaire that evaluates cognitive fluctuations during the previous month [125]. It allows a shorter version called Mayo Fluctuations Composite Score (4-item scale), which is useful for the detection of cognitive fluctuations but does not determine their severity [125]. The One Day Fluctuations Assessment Scale (7-item scale) correlates with neuropsychological and electrophysiological measures of cognitive fluctuations [123]. However, the evaluation is limited to the last 24-h and it includes features that are not specific to DLB, reducing its sensitivity [122, 126]. Finally, the Dementia Cognitive Fluctuation Scale (17-item questionnaire) was derived from the previous scales and captures multiple aspects of cognitive fluctuations with good validity and reliability although it is lengthy to administer, thus limiting its utility [127] (Additional file 2: Table S2).

In addition to the scales described above, the use of neuropsychological tests that provide information on the variability of response times may also be considered as a surrogate marker for cognitive fluctuations. Using computerized batteries, such as the sustained attention response task, choice reaction time, simple reaction time, or digit vigilance, the degree of variability in the response has been shown to correlate with performance in clinical cognitive fluctuation scales without being affected by motor deficits [126, 128]. However, whilst these tools show promise in the evaluation of response to medications impacting cognitive fluctuations, they have yet to be validated. Following the same principle, electroencephalography (EEG) has shown promise in the detection of cognitive fluctuations, manifesting as changes in variability in the dominant frequency band. Such changes have a good correlation with the CAF and neuropsychological measures of attentional variability [129, 130]. Further validation of EEG measures as surrogate markers for cognitive fluctuations, including portable EEG, is warranted.

Excessive daytime sleepiness (EDS) is a separate clinical feature, supportive of the diagnosis of DLB and its significant impact on daily functioning and caregiver burden, making it an important target for therapies [13, 131]. While EDS is considered to be closely related to cognitive fluctuations as well as cognition and sleep disturbances in DLB, further studies are needed to define these relationships [126]. EDS is included as part of the Mayo Fluctuation Scale [125]. However, specific scales, such as the Epworth Sleepiness Scale, which have been used in trials targeting EDS in DLB and PD, are available and may be preferable if EDS is considered an outcome [62, 132, 133].

We propose that the choice of the assessment scale used to evaluate cognitive fluctuations will depend on whether the goal of the study is to identify their presence as a clinical milestone or measure their frequency/severity and over what time period. Alternatively, if the goal is to assess the presence of cognitive fluctuations as a potential confounder of cognitive performance, then scales sensitive to the detection of fluctuations could be utilized along with computerized testing. Further evaluation of the measurement properties of all of the available scales measuring fluctuations is warranted. In addition, more consideration should be given to validating objective vigilance tasks (e.g., Choice Reaction Time, Sustained Attention to Response Task, etc.) that could also be used at the beginning and end of cognitive assessments to capture any significant variability in performance. Finally, the assessment of EDS could be used to complement the evaluation of cognitive fluctuations or be considered as an individual endpoint, depending on the goal of the trial.

### Visual hallucinations

Neuropsychiatric symptoms are common in DLB and recurrent visual hallucinations (VH) deserve particular attention given that they occur in up to 80% of patients and form one of the core criteria for diagnosis. The presence and severity of VH can be assessed either through composite scales that include at least one question focusing on hallucinations (e.g., MDS-UPDRS, neuropsychiatric inventory-NPI), hallucination scales covering other modalities (e.g., auditory), or scales specifically for VH. Scoring for VH can typically be frequency-based, severity-based, or can capture both frequency and severity. Frequency-based scoring is easier to administer but can be subject to recall and perception bias. Both frequency- and severity-based scores have been used in clinical trials, although severity may have greater clinical relevance. However, deciding what severity aspect of the phenomena (e.g., intensity, emotional reaction) should be measured as an outcome has not been determined.

The Neuropsychiatric Inventory (NPI) is a structured caregiver interview that was originally designed to detect, quantify and track neuropsychiatric symptom changes in people with dementia [134]. The NPI has shown good content validity, concurrent validity, inter-rater reliability, and test–retest reliability in patients with dementia [135]. Some of the other validated and widely used versions of NPI are the Nursing Home (NPI—NH)[136] and the Brief Form (NPI-Q, validated in AD patients)[137]. When it comes to DLB, it is important to emphasize that the NPI covers hallucinations in all modalities (e.g., visual, auditory) under the same question, which may significantly affect the evaluation of visual hallucinations in clinical trials. The hallucination questions have been used in one DLB trial, while the combination of the hallucination and delusion questions has been used as an outcome for one PD trial, showing that a combination is responsive to treatment effects [60, 138]. However, it has not been validated for DLB, nor an MCID determined. The Scale for the Assessment of Positive Symptoms for Parkinson's Disease Psychosis (SAPS-PD) evaluates multiple modalities of hallucinations and includes other elements of psychosis, such as delusions. However, SAPS-PD was not developed as a tool for measuring change, which affects its reliability, although it has been used this way in treatment trials for PD psychosis [139, 140]. Another scale that includes multiple neuropsychiatric symptoms is the Behavioral Pathology in Alzheimer's Disease Rating Scale (BEHAVE-AD), which covers multiple aspects of neuropsychiatric symptoms but is particularly focused on AD [141]. This original version of this scale focused on AD, including five questions covering the different modalities of hallucinations with only one question on the severity rather than frequency of VH. However, a newer version of the scale including the frequency of VH has subsequently been developed [142]. The VH question from the BEHAVE-AD has been used in one DLB trial to evaluate the benefit of rivastigmine in VH, which demonstrated its sensitivity to treatment effects [94]. Both the UPDRS and MDS-UPDRS include a single question on VH, which is combined with other features of psychosis such as delusions. This approach has been used in one DLB trial showing that increases in levodopa may help motor symptoms at the cost of worsening psychotic symptoms [143].

Regarding multimodal hallucination scales, the University of Miami Parkinson's disease Hallucinations Questionnaire is easy to administer and a useful tool for capturing both the severity and frequency of VH [144]. Although its responsiveness to change has not yet been fully validated, it is in use in a current treatment trial for VH in PDD and DLB [145]. The Psychosis and Hallucinations Questionnaire offers a patient and informant self-report approach but to date has only been validated in non-demented PD [146, 147]. The North-East Visual Hallucination Interview (NEVHI) was designed to specifically assess visual-domain hallucinations and utilizes a semi-structured interview focusing on different aspects that affect the severity of VH. Further, this scale has both informant and patient versions which might allow for enhanced accuracy in terms of frequency. The NEVHI has shown to be a valid and reliable scale with a strong correlation with the MDS-UPDRS hallucination item score, making it a promising scale for trials focusing on VH [148, 149] (Additional file 3: Table S3).

Potential alternatives to interviews and questionnaires are tasks eliciting visual misperceptions, such as the Pareidolia Test and the Bistable Percept Paradigm, which have been suggested as surrogate markers for VH [150–153]. However, their sensitivity and specificity appear to be low within the early stages of the disease, and it is unclear whether these measures are suited for measuring change in a meaningful way over time [154].

We recommend that the choice of the VH scale will depend on the goal of the trial. Hallucination-specific scales may be preferred in trials focusing on symptomatic treatment of VH or DMTs where the progression of the disease is associated with the onset of VH. Instead, the use of broader neuropsychiatric scales and questionnaires that can capture other common features such as depression, anxiety, and apathy should be considered.

### Motor outcomes

Motor changes in DLB mostly fall under the umbrella of the parkinsonian syndrome, which includes slowness, tremor, and rigidity. The severity of these features varies among DLB patients and may be absent in up to 15% of cases [13]. While dopamine depletion may be the underlying cause for parkinsonism in DLB, each one of the cardinal symptoms can develop through different mechanisms. This is important because a benefit in one symptom may lead to the worsening of another one, such as cholinergic effects potentially improving gait but worsening tremor [143, 155].

Motor scales used for DLB are based on scales developed for PD, which might be problematic since the motor syndrome in DLB differs from that in PD [13]. The motor sections of the UPDRS and the MDS-UPDRS, also known as Part III, offer the possibility of evaluating the main features of parkinsonism. The UPDRS is responsive to therapeutic interventions and is the reference scale for regulatory agencies [156, 157]. Moreover, it has been used as a primary outcome in DLB trials targeting motor function [99, 158]. The MDS-UPDRS is a newer version that differs from the UPDRS especially in the evaluation of non-motor aspects of PD [27]. In addition, the MDS-UPDRS adds further motor aspects, including freezing of gait, separating postural and kinetic tremor, and separating the amplitude and constancy of rest tremor. In addition, the MDS-UPDRS score can be converted to the former UPDRS assessments [159]. The main drawback is that by measuring the composite score as a whole, an improvement in one domain can be masked by deficits in another. This is particularly important when it comes to using these scales, where tremor amplitude constitutes a significant portion of the total score compared to other motor domains such as postural reflexes, which can be more relevant for daily functioning [160]. Another drawback of both the UPDRS and the MDS-UPDRS is the time needed to administer the scale and the experience of the rater, limiting its use in multi-site trials (Additional file 4: Table S4). Among the motor changes in DLB, impairment in balance and falls are the most relevant. Falling in DLB is associated with substantial morbidity and mortality [161]. While both versions of the UPDRS include an evaluation of gait and balance, they do not include a measurement of the number of falls.

Many TOMs are also being developed and may represent an option to complement the motor assessment [162]. TOMs may allow the capture of both motor and non-motor phenomena with greater accuracy and reduced intra- and inter-rater variability than clinical scales for a continuous period of time with good temporal resolution [162]. However, little is known about whether this information represents a meaningful measure of the patients’ activities of daily living or functional activity [163]. Within the TOMs exist tools that allow kinematic analysis, such as gait mats and infrared cameras, along with wearable sensors, such as gyroscopes or accelerometers. These technologies hold the promise of improving sensitivity, accuracy, reproducibility, and feasibility of objectively capturing changes in motor function, especially when assessed at home in unsupervised conditions [164, 165]. However, their implementation in clinical trials of parkinsonism remains at an early stage. The main limitations of TOMs are their lack of validation outside the clinic and the requirement for specific pieces of equipment, which may not be widely available. In DLB, the presence of cognitive impairment and fluctuations may also limit the compliance of TOMS, and patient/caregiver technology surveys are still lacking.

For evaluation of motor features of DLB, we suggest using the validated UPDRS scales to measure parkinsonism and incorporating a survey for falls. Further consideration would need to be given to the medication state of the patient and whether standardizing assessments in the ‘off’ dopaminergic medication state would be required. The use of TOMs will require their further validation.

### Rapid eye movement sleep behavior disorder

Rapid eye movement (REM) sleep behavior disorder (RBD) is highly prevalent and is the final core clinical feature in the diagnosis of DLB [13]. In addition, given that isolated RBD is the most reliable clinical marker of prodromal synucleinopathies, its detection and the assessment of its severity are of particular importance for DMTs [166]. Assessment of RBD can be done through scales or objectively through video polysomnography (vPSG).

Scales provide an obvious advantage when it comes to the time needed from the research patient and the cost of trials. However, the reliance on patients' and bedpartners' recall regarding symptoms that occur during sleep makes scales less desirable as outcome measures rather than for their use as screening tools [167]. The REM Sleep Behavior Disorder Screening Questionnaire is commonly used as a screening test but it does not provide much information on the severity of symptoms nor has its sensitivity to change been studied [168]. The REM Sleep Behavior Disorder Questionnaire Hong Kong is used to evaluate the presence of RBD and also quantifies the frequency and severity of dream enactment [169]. This scale has shown good validity and reliability. In addition, its sensitivity to change after treatment was previously demonstrated across multiple studies [170, 171]. However, the English version of this tool is not yet validated. The Mayo Sleep Questionnaire (MSQ) has been evaluated in patients with cognitive impairment where it has been shown to be adequate for the detection of RBD [172]. In addition, the REM Sleep Behavior Disorder Single-Question Screen, a screening question for dream enactment with a simple yes/no response, has also shown appropriate sensitivity and specificity [173]. Among the questionnaires described above, only the MSQ is based on responses from an informant, while the others are based on responses from the patient; considering that cognitive impairment is present in those with DLB, an informant-based questionnaire is potentially more valid. The REM sleep behavior disorder symptom severity scale, which measures the frequency and severity of RBD features over a defined period of time, as reported by the patient and the informant, is currently undergoing reliability and validation analyses (unpublished data).

Objectively, RBD can be quantified by vPSG using the RBD Severity Scale to quantify the occurrence and severity of RBD [174]. Multiple methods have already been developed for the quantification of REM sleep without atonia (RSWA) but current data are insufficient to support RSWA as a dynamic marker in DLB [174]. Due to the variability in RBD symptom severity from night to night, two or more nights of vPSG have been recommended to improve any effect size. However, such an approach would pose a major challenge to the feasibility of any future trials focused on this symptom [175]. It must also be appreciated that laboratory-based vPSG represents a potentially biased snapshot with patients sleeping in unfamiliar surroundings [174] (Additional file 5: Table S5). Furthermore, there is no evidence to suggest that vPSG is superior to any clinical scales such as sleep diaries. Considering that there can be night-to-night variability in RBD frequency and severity, the use of vPSG over a few nights before beginning a therapy and again weeks or months later is debatable.

Although home-based PSG devices are emerging as a promising alternative, some cannot currently ascertain whether the patient is in REM sleep. Furthermore, others do not assess EMG tone of skeletal muscles, and therefore their sensitivity to treatment has not yet been demonstrated [174, 176]. However, recent data suggest that some devices warrant further study for detecting and monitoring RBD [177]. In addition, activity sensors, such as actigraphy, have been proposed as an objective outcome for trials of RBD, along with automated video analysis of the leg movements during REM sleep [176]. Home-based actigraphy to characterize sleep disturbances that cannot be captured by conventional clinical scales may be more ecologically valid than PSG [162]. Complementing home-based measurement with sleep diaries may be a more pragmatic approach for clinical trials of DLB in the near future [176, 178].

We recommend that the choice of RBD assessment will likely need to focus on the presence and severity of RBD behaviors and the impact of RBD on everyday life. While objective measures are preferable, their applicability may hinder the feasibility of the study, and scales assessing the impact of RBD should complement these objective measures. The validation of portable technologies, such as actigraphy, will open the door to much needed accessible, robust, and objective outcome measures.

### Composite outcomes

Increasingly, composite scales, particularly those that integrate both functional and cognitive dimensions, have been used as primary endpoints in AD trials. This approach has been favored in the preclinical, prodromal, and mild AD trials to replace the use of the previously required co-primary outcomes (i.e., one cognitive and one functional outcome) [179, 180]. Co-primary outcomes were considered to be too strict for use in pre-dementia trials where few longitudinal changes occur over the course of the trials. However, concerns about their ability to capture change and their validity have been raised [179]. The Clinical Dementia Rating Scale Sum of Boxes (CDR-SOB) has been validated for use in both AD and PD and has been used as a primary outcome in trials of potential DMTs [181]. In PDD, the CDR-SOB showed a good correlation with activities of daily living and cognitive performance [182]. Furthermore, it has been proposed that the Integrated AD Rating Scale, comprising the ADAS-Cog and a scale of activities of daily living (ADCS-iADL), may provide a better signal-to-noise ratio than the CDR-SOB [183, 184]. The Cognitive-Functional Composite (CFC) has also emerged as a useful scale due to its feasibility, validity, and reliability, as well as clinical relevance in AD and has been used in clinical trials [185, 186]. However, the CFC has only been used in a small number of patients with DLB, limiting the conclusions on its applicability in DLB clinical trials [186]. We are not aware of composite scores including RBD, autonomic function, motor deficits, and cognition yet developed in DLB (Table 6).

**Table 6:** Selected composite outcomes

Outcome	Domains	Reliability	Responsiveness	MCID	Used in DLB trials
CDR-SOB	Cog, ADL	+/−	+	NE	Yes [60, 82]
ADS-iADL	Cog, ADL	+	+	NE	No
CFC	Cog, ADL	+	NE	NE	No

*ADL* Activities of daily living measure, *Cog* cognitive measure, *MCID* minimal clinically important difference, *NE* not evaluated, *CDR-SOB* Clinical Dementia Rating Scale Sum of Boxes, *ADS-iADl* Alzheimer’s Disease Assessment Scale Activities of Daily Living, *CFC* Cognitive Functional Composite

+, good/adequate; +/−, acceptable; −, performance is questionable/mediocre

## Conclusions

The selection of robust and valid outcome measures is important when designing any clinical trial. However, the wide variety of symptoms and multiple clinical domains poses challenges for clinical trials in DLB. We suggest that all domains are evaluated in clinical trials with a specific emphasis depending on the intended target of the intervention (i.e., symptomatic or DMT).

The choice of the primary outcome depends on the goal of the therapeutic intervention being evaluated and the stage and severity of the population studied. Symptomatic-treatment trials may choose to focus on a functional or global impression of change as a primary outcome with a secondary domain-specific outcome. In the case of DMTs, the choice of the outcome varies depending on the severity of the population studied. Trials focusing on prodromal or preclinical groups may choose the emergence of a clinical feature of DLB (e.g., dementia or VH) or overall function or cognition as a primary outcome. Further understanding of the progression of the different domains affected by DLB at each clinical stage of disease will be crucial to defining appropriate outcome measures [187, 188]. A different approach will likely be needed in trials focusing on diagnosed DLB patients, where the severity of core clinical features may be a more suitable outcome. In any case, those that are not considered primary or secondary outcomes may be evaluated with measures used to detect their presence for diagnostic classification.

Currently, there is a lack of DLB-specific validated outcome measures. A bespoke clinical global outcome scale that not only reflects a composite of cognitive and functional impairment but also includes the contribution of other DLB symptoms, would represent the ideal primary endpoint for DLB trials, particularly those evaluating DMTs. The development of a DLB-specific outcome measure will need to be tailored to the type of trial in which it will be used (i.e., symptomatic vs disease-modifying therapies) and the stage of the disease being investigated (e.g., prodromal, early, advanced). The process of scale development is a rigorous process that requires multiple steps, and the development of a novel outcome measure may be more time- and labor-intensive than the validation of an existing one. Existing outcome measures developed for conditions such as AD or PD may be useful for DLB. However, they require validation in the DLB population and determination of their measurement properties, including responsiveness. Moreover, increasing attention has been paid to the involvement of patients and caregivers in clinical trial design, which also applies to clinical outcome measures [189]. A limitation of our review is the absence of patient and caregiver input. Future outcome measure initiatives need to include DLB patient advocate groups in the development phase to gain perspectives on important clinical outcomes for patients and caregivers. In addition, feedback from regulatory agencies early in the development process is advised. Finally, determining the MCID for these measures and for TOMs will guide the design of the trials and their interpretation (Table 7). This will lead to better-powered clinical trials and, ultimately, to more rapid progress towards therapeutic interventions providing meaningful clinical improvement in patients with DLB.

**Table 7:** Selected potentially useful technology-based objective measures

Technology	Potential applications as outcome measures
Tablets and smartphones	Cognitive testing
	Cognitive fluctuations
	Sustained attention
	Response task
	Frequent completion of questionnaires in different domains
	Motor function (plotting speed and accuracy)
Tracking devices	Functional
	Activity
	Cognitive
	Wandering
	Motor
	Activity
	Falls
Wearable sensors (e.g., gyroscope, accelerometers, heart rate monitors, etc.)	Functional
	Activity
	Motor
	Parkinsonism
	Gait
	Falls
	RBD/sleep/EDS
	Autonomic
	Heart rate variability
	Blood pressure
Kinematic analysis (e.g., gait mats, cameras, etc.)	Functional
	Home sensor monitoring of activities
	Motor
	Gait
	Falls
	RBD
Home sensor monitoring	Functional
	Monitor use of appliances
Portable EEG	Cognitive fluctuation
	Sleep
	RBD
Virtual reality	Functional
	ADL simulator
	Use of appliances
	Driving simulator
	Financial simulator

*RBD* REM Sleep Behavior Disorder, *ADL* Activities of Daily Living, *EDS* Excessive Daytime Sleepiness, *EEG* Electroencephalogram

## Supplementary Information

**Additional file 1. Table S1**: Cognitive and neuropsychological measures utilized in DLB clinical trials.

**Additional file 2. Table S2**: Selected cognitive fluctuations outcomes.

**Additional file 3. Table S3**: Selected visual hallucinations outcomes.

**Additional file 4. Table S4**: Selected motor outcomes.

**Additional file 5. Table S5**: Selected REM-sleep behavior disorder outcomes.

## Data Availability

Not applicable.

## Abbreviations

AD: Alzheimer's disease
ADAS-Cog: Alzheimer’s Disease Assessment Scale-Cognitive
ADCS-ADL: Alzheimer’s Disease Cooperative Study-Activities of Daily Living Scale
ADCS-CGIC: Alzheimer’s Disease Cooperative Study–Clinician’s Global Impression of Change
BEHAVE-AD: Behavioral Pathology in Alzheimer's Disease Rating Scale
CAF: Clinician Assessment of Fluctuation
CDR-SOB: Clinical Dementia Rating-Sum of Boxes
CFC: Cognitive-Functional Composite
CGI: Clinical Global Impression Scale
DAD: Disability Assessment for Dementia
DLB: Dementia with Lewy bodies
DMT: Disease-modifying therapy
EDS: Excessive daytime sleepiness
EEG: Electroencephalography
MCI: Mild cognitive impairment
MCID: Minimal clinically important difference
MDRS: Mattis Dementia Rating Scale
MDS-UPDRS: Movement Disorders Society Unified Parkinson's Disease Rating Scale
MMSE: Mini-Mental State Examination
MSQ: Mayo Sleep Questionnaire
NEVHI: North-East Visual Hallucination Interview
NPI: Neuropsychiatric Inventory
NPI-Q: Neuropsychiatric Inventory-Questionnaire
PD: Parkinson disease
PDD: Parkinson disease dementia
PDQ-39: 39-Item Parkinson’s Disease Questionnaire
QOL-AD: Quality of Life in Alzheimer’s Disease Scale
RBD: Rapid eye movement sleep behavior disorder
REM: Rapid eye movement
RSWA: REM sleep without atonia
SAPS-PD: Scale for the Assessment of Positive Symptoms for Parkinson's Disease Psychosis
TOMs: Technology-based objective measures
UPDRS: Unified Parkinson's Disease Rating Scale
VH: Visual hallucinations
vPSG: Video polysomnography
ZBI: Zarit Burden Interview
