# Correction: Clinical outcome measures in dementia with Lewy bodies trials: critique and recommendations

**DOI:** 10.1186/s40035-022-00306-0

**Published:** 2022-05-17

**Authors:** Federico Rodriguez-Porcel, Kathryn A. Wyman-Chick, Carla Abdelnour Ruiz, Jon B. Toledo, Daniel Ferreira, Prabitha Urwyler, Rimona S. Weil, Joseph Kane, Andrea Pilotto, Arvid Rongve, Bradley Boeve, John-Paul Taylor, Ian McKeith, Dag Aarsland, Simon J. G. Lewis, the Lewy Body Dementias Clinical Trials Workgroup from the Lewy Body Dementias Professional Interest Area - Alzheimer’s Association International Society to Advance Alzheimer’s Research and Treatment (ISTAART)+

**Affiliations:** 1 Department of Neurology, Medical University of South Carolina, 208b Rutledge Av., Charleston, SC 29403 USA; 2 Department of Neurology, Center for Memory and Aging, HealthPartners, Saint Paul, MN USA; 3 Autonomous University of Barcelona, Barcelona, Spain; 4 Fixel Institute for Neurological Diseases, University of Florida, Gainesville, FL USA; 5 Division of Clinical Geriatrics, Department of Neurobiology, Care Sciences, and Society, Center for Alzheimer’s Research, Karolinska Institutet, Stockholm, Sweden; 6 Department of Radiology, Mayo Clinic, Rochester, MN USA; 7 ARTORG Center for Biomedical Engineering Research, University of Bern, Bern, Switzerland; 8 Dementia Research Centre, University College London, London, UK; 9 Centre for Public Health, Queen’s University, Belfast, UK; 10 Neurology Unit, Department of Clinical and Experimental Sciences, University of Brescia, Brescia, Italy; 11 Department of Research and Innovation, Helse Fonna, Haugesund Hospital, Haugesund, Norway; 12 Institute of Clinical Medicine (K1), The University of Bergen, Bergen, Norway; 13 Department of Neurology, Center for Sleep Medicine, Mayo Clinic, Rochester, MN USA; 14 Translational and Clinical Research Institute, Newcastle University, Newcastle upon Tyne, UK; 15 Department of Old Age Psychiatry Institute of Psychiatry Psychology and Neuroscience, King’s College London, London, UK; 16 ForeFront Parkinson’s Disease Research Clinic, Brain and Mind Centre, School of Medical Sciences, University of Sydney, 100 Mallett Street, Camperdown, NSW 2050 Australia

## Correction to: Translational Neurodegeneration (2022) 11:24 https://doi.org/10.1186/s40035-022-00299-w

Following publication of the original article [1], the authors identified some errors in the Additional files 1–5.

The original article [1] has been corrected.
