# Supplementary material for: Clinical outcome measures in dementia with Lewy bodies trials: critique and recommendations
Source: Transl Neurodegener. 2022 May 2;11:24. doi: 10.1186/s40035-022-00299-w (PMC9059356; doi:10.1186/s40035-022-00299-w)
Supplement: Supplementary file 1 — Additional file 1. Table S1: Cognitive and neuropsychological measures utilized in DLB clinical trials. [file 40035_2022_299_MOESM1_ESM.docx]

**Supplemental Table 1. Selected cognitive fluctuations outcomes**

| Outcome | Rater | Detection | Discrimination | Reliability | Responsiveness | Used in trials |
| --- | --- | --- | --- | --- | --- | --- |
| CAF | Clinician | +* | +* | + | NE | Yes[1] |
| Mayo FS | Informant | +* | NE | + | NE | Yes[2-5] |
| ODFAS | Informant | +/-* | +* | NE | NE | Yes[1, 6, 7] |
| DCFS | Informant | +* | +* | + | NE | No |

+, good/adequate; +/-, acceptable performance is questionable/mediocre. MCID: minimal clinically important difference; NE: Not evaluated. * Evaluated in DLB population. Clinician Assessment of Fluctuation; Mayo FS = Mayo Fluctuation Scale; ODFAS = One Day Fluctuations Assessment Scale; DCFS = Dementia Cognitive Fluctuation Scale.

**REFERENCES**

1. Levin OS, Batukaeva LA, Smolentseva IG, Amosova NA. Efficacy and safety of memantine in Lewy body dementia. Neurosci Behav Physiol. 2009;39(6):597-604.

2. Ikeda M, Mori E, Kosaka K, Iseki E, Hashimoto M, Matsukawa N, et al. Long-term safety and efficacy of donepezil in patients with dementia with Lewy bodies: results from a 52-week, open-label, multicenter extension study. Dement Geriatr Cogn Disord. 2013;36(3-4):229-41.

3. Gratwicke J, Zrinzo L, Kahan J, Peters A, Brechany U, McNichol A, et al. Bilateral nucleus basalis of Meynert deep brain stimulation for dementia with Lewy bodies: A randomised clinical trial. Brain Stimul. 2020;13(4):1031-9.

4. Thomas AJ, Burn DJ, Rowan EN, Littlewood E, Newby J, Cousins D, et al. A comparison of the efficacy of donepezil in Parkinson's disease with dementia and dementia with Lewy bodies. Int J Geriatr Psychiatry. 2005;20(10):938-44.

5. Rowan E, McKeith IG, Saxby BK, O'Brien JT, Burn D, Mosimann U, et al. Effects of donepezil on central processing speed and attentional measures in Parkinson's disease with dementia and dementia with Lewy bodies. Dement Geriatr Cogn Disord. 2007;23(3):161-7.

6. Elder GJ, Colloby SJ, Firbank MJ, McKeith IG, Taylor JP. Consecutive sessions of transcranial direct current stimulation do not remediate visual hallucinations in Lewy body dementia: a randomised controlled trial. Alzheimers Res Ther. 2019;11(1):9.

7. Edwards K, Royall D, Hershey L, Lichter D, Hake A, Farlow M, et al. Efficacy and safety of galantamine in patients with dementia with Lewy bodies: a 24-week open-label study. Dement Geriatr Cogn Disord. 2007;23(6):401-5.
