# Supplementary material for: Clinical outcome measures in dementia with Lewy bodies trials: critique and recommendations
Source: Transl Neurodegener. 2022 May 2;11:24. doi: 10.1186/s40035-022-00299-w (PMC9059356; doi:10.1186/s40035-022-00299-w)
Supplement: Supplementary file 2 — Additional file 2. Table S2: Selected cognitive fluctuations outcomes. [file 40035_2022_299_MOESM2_ESM.docx]

**Supplemental Table 2. Selected visual hallucinations outcomes**

| Outcome | Domains | Reliability | Responsiveness | MCID | Used in trials |
| --- | --- | --- | --- | --- | --- |
| NPI | NPS | + | Yes | 8 points | Yes[1-21] |
| BEHAVE-AD | NPS | + | Yes | NE | Yes[20, 22-24] |
| SAPS-PD | Psychosis | NE | NE | NE | Yes[25] |
| PsycH-Q | Psychosis | NE | NE | NE | No |
| UMPDHQ | Hallucinations | + | NE | NE | No |
| NEVHI | VH | + | NE | NE | Yes[26] |

+, good/adequate; +/-, acceptable performance is questionable/mediocre. MCID: minimal clinically important difference; NE: not evaluated; NPS: neuropsychiatric symptoms; VH: visual hallucinations. NPI: Neuropsychiatric Inventory; BEHAVE-AD: Behavioral Pathology in Alzheimer's Disease Rating Scale; SAPS-PD: Scale for the Assessment of Positive Symptoms for Parkinson's Disease Psychosis; PsycH-Q: Psychosis and Hallucinations Questionnaire; UMPDHQ: University of Miami Parkinson's disease Hallucinations Questionnaire; NEVHI: North-East Visual Hallucination Interview.

**REFERENCES**

1. Mori E, Ikeda M, Kosaka K. Donepezil for dementia with Lewy bodies: a randomized, placebo-controlled trial. Ann Neurol. 2012;72(1):41-52.

2. Thomas AJ, Burn DJ, Rowan EN, Littlewood E, Newby J, Cousins D, et al. A comparison of the efficacy of donepezil in Parkinson's disease with dementia and dementia with Lewy bodies. Int J Geriatr Psychiatry. 2005;20(10):938-44.

3. Minett TS, Thomas A, Wilkinson LM, Daniel SL, Sanders J, Richardson J, et al. What happens when donepezil is suddenly withdrawn? An open label trial in dementia with Lewy bodies and Parkinson's disease with dementia. Int J Geriatr Psychiatry. 2003;18(11):988-93.

4. Mori E, Ikeda M, Nakagawa M, Miyagishi H, Yamaguchi H, Kosaka K. Effects of Donepezil on Extrapyramidal Symptoms in Patients with Dementia with Lewy Bodies: A Secondary Pooled Analysis of Two Randomized-Controlled and Two Open-Label Long-Term Extension Studies. Dement Geriatr Cogn Disord. 2015;40(3-4):186-98.

5. McKeith I, Del Ser T, Spano P, Emre M, Wesnes K, Anand R, et al. Efficacy of rivastigmine in dementia with Lewy bodies: a randomised, double-blind, placebo-controlled international study. Lancet. 2000;356(9247):2031-6.

6. McKeith IG, Grace JB, Walker Z, Byrne EJ, Wilkinson D, Stevens T, et al. Rivastigmine in the treatment of dementia with Lewy bodies: preliminary findings from an open trial. Int J Geriatr Psychiatry. 2000;15(5):387-92.

7. Mizukami K, Asada T, Kinoshita T, Tanaka K, Sonohara K, Nakai R, et al. A randomized cross-over study of a traditional Japanese medicine (kampo), yokukansan, in the treatment of the behavioural and psychological symptoms of dementia. Int J Neuropsychopharmacol. 2009;12(2):191-9.

8. Edwards K, Royall D, Hershey L, Lichter D, Hake A, Farlow M, et al. Efficacy and safety of galantamine in patients with dementia with Lewy bodies: a 24-week open-label study. Dement Geriatr Cogn Disord. 2007;23(6):401-5.

9. Kimura T, Hayashida H, Murata M, Takamatsu J. Effect of ferulic acid and Angelica archangelica extract on behavioral and psychological symptoms of dementia in frontotemporal lobar degeneration and dementia with Lewy bodies. Geriatr Gerontol Int. 2011;11(3):309-14.

10. Mori S, Mori E, Iseki E, Kosaka K. Efficacy and safety of donepezil in patients with dementia with Lewy bodies: preliminary findings from an open-label study. Psychiatry Clin Neurosci. 2006;60(2):190-5.

11. Ikeda M, Mori E, Kosaka K, Iseki E, Hashimoto M, Matsukawa N, et al. Long-term safety and efficacy of donepezil in patients with dementia with Lewy bodies: results from a 52-week, open-label, multicenter extension study. Dement Geriatr Cogn Disord. 2013;36(3-4):229-41.

12. Aarsland D, Ballard C, Walker Z, Bostrom F, Alves G, Kossakowski K, et al. Memantine in patients with Parkinson's disease dementia or dementia with Lewy bodies: a double-blind, placebo-controlled, multicentre trial. Lancet Neurol. 2009;8(7):613-8.

13. Emre M, Tsolaki M, Bonuccelli U, Destée A, Tolosa E, Kutzelnigg A, et al. Memantine for patients with Parkinson's disease dementia or dementia with Lewy bodies: a randomised, double-blind, placebo-controlled trial. Lancet Neurol. 2010;9(10):969-77.

14. Lucetti C, Logi C, Del Dotto P, Berti C, Ceravolo R, Baldacci F, et al. Levodopa response in dementia with lewy bodies: a 1-year follow-up study. Parkinsonism Relat Disord. 2010;16(8):522-6.

15. Molloy SA, Rowan EN, O'Brien JT, McKeith IG, Wesnes K, Burn DJ. Effect of levodopa on cognitive function in Parkinson's disease with and without dementia and dementia with Lewy bodies. J Neurol Neurosurg Psychiatry. 2006;77(12):1323-8.

16. Iwasaki K, Kosaka K, Mori H, Okitsu R, Furukawa K, Manabe Y, et al. Open label trial to evaluate the efficacy and safety of Yokukansan, a traditional Asian medicine, in dementia with Lewy bodies. J Am Geriatr Soc. 2011;59(5):936-8.

17. Lapid MI, Kuntz KM, Mason SS, Aakre JA, Lundt ES, Kremers W, et al. Efficacy, Safety, and Tolerability of Armodafinil Therapy for Hypersomnia Associated with Dementia with Lewy Bodies: A Pilot Study. Dement Geriatr Cogn Disord. 2017;43(5-6):269-80.

18. Cummings JL, Street J, Masterman D, Clark WS. Efficacy of olanzapine in the treatment of psychosis in dementia with lewy bodies. Dement Geriatr Cogn Disord. 2002;13(2):67-73.

19. Murata M, Odawara T, Hasegawa K, Iiyama S, Nakamura M, Tagawa M, et al. Adjunct zonisamide to levodopa for DLB parkinsonism: A randomized double-blind phase 2 study. Neurology. 2018;90(8):e664-e72.

20. Culo S, Mulsant BH, Rosen J, Mazumdar S, Blakesley RE, Houck PR, et al. Treating neuropsychiatric symptoms in dementia with Lewy bodies: a randomized controlled-trial. Alzheimer Dis Assoc Disord. 2010;24(4):360-4.

21. McCormick SA, Vatter S, Carter LA, Smith SJ, Orgeta V, Poliakoff E, et al. Parkinson's-adapted cognitive stimulation therapy: feasibility and acceptability in Lewy body spectrum disorders. J Neurol. 2019;266(7):1756-70.

22. Walker Z, Grace J, Overshot R, Satarasinghe S, Swan A, Katona CL, et al. Olanzapine in dementia with Lewy bodies: a clinical study. Int J Geriatr Psychiatry. 1999;14(6):459-66.

23. Samuel W, Caligiuri M, Galasko D, Lacro J, Marini M, McClure FS, et al. Better cognitive and psychopathologic response to donepezil in patients prospectively diagnosed as dementia with Lewy bodies: a preliminary study. Int J Geriatr Psychiatry. 2000;15(9):794-802.

24. Satoh M, Ishikawa H, Meguro K, Kasuya M, Ishii H, Yamaguchi S. Improved visual hallucination by donepezil and occipital glucose metabolism in dementia with Lewy bodies: the Osaki-Tajiri project. Eur Neurol. 2010;64(6):337-44.

25. McKeith I, Aarsland D, Friedhoff L, Lombardo I, France N, Dworak H, et al. HEADWAY-DLB: A multinational study evaluating the safety and efficacy of intepirdine (rvt-101) in dementia with lewy bodies. Alzheimer's & Dementia. 2017;13:P936.

26. Gratwicke J, Zrinzo L, Kahan J, Peters A, Brechany U, McNichol A, et al. Bilateral nucleus basalis of Meynert deep brain stimulation for dementia with Lewy bodies: A randomised clinical trial. Brain Stimul. 2020;13(4):1031-9.
