# Supplementary material for: Clinical outcome measures in dementia with Lewy bodies trials: critique and recommendations
Source: Transl Neurodegener. 2022 May 2;11:24. doi: 10.1186/s40035-022-00299-w (PMC9059356; doi:10.1186/s40035-022-00299-w)
Supplement: Supplementary file 3 — Additional file 3. Table S3: Selected visual hallucinations outcomes. [file 40035_2022_299_MOESM3_ESM.docx]

**Supplemental Table 3. Selected motor outcomes**

| Outcome | Reliability | Responsiveness | MCID | Used in trials |
| --- | --- | --- | --- | --- |
| UPDRS | + | + | NE | Yes[1-23] |
| MDS-UPDRS | + | + | NE | Yes[24-26] |

+, good/adequate; +/-, acceptable performance is questionable/mediocre. MCID: minimal clinically important difference; NE: not evaluated. UPDRS = Unified Parkinson’s Disease Rating Scale; MDS-UPDRS = Movement Disorder Society Sponsored Revision of the Unified Parkinson’s Disease Rating Scale.

**REFERENCES**

1. McKeith I, Del Ser T, Spano P, Emre M, Wesnes K, Anand R, et al. Efficacy of rivastigmine in dementia with Lewy bodies: a randomised, double-blind, placebo-controlled international study. Lancet. 2000;356(9247):2031-6.

2. Pagan F, Hebron M, Valadez EH, Torres-Yaghi Y, Huang X, Mills RR, et al. Nilotinib Effects in Parkinson's disease and Dementia with Lewy bodies. J Parkinsons Dis. 2016;6(3):503-17.

3. Murata M, Odawara T, Hasegawa K, Iiyama S, Nakamura M, Tagawa M, et al. Adjunct zonisamide to levodopa for DLB parkinsonism: A randomized double-blind phase 2 study. Neurology. 2018;90(8):e664-e72.

4. Walker Z, Grace J, Overshot R, Satarasinghe S, Swan A, Katona CL, et al. Olanzapine in dementia with Lewy bodies: a clinical study. Int J Geriatr Psychiatry. 1999;14(6):459-66.

5. Minett TS, Thomas A, Wilkinson LM, Daniel SL, Sanders J, Richardson J, et al. What happens when donepezil is suddenly withdrawn? An open label trial in dementia with Lewy bodies and Parkinson's disease with dementia. Int J Geriatr Psychiatry. 2003;18(11):988-93.

6. Molloy S, McKeith IG, O'Brien JT, Burn DJ. The role of levodopa in the management of dementia with Lewy bodies. J Neurol Neurosurg Psychiatry. 2005;76(9):1200-3.

7. Thomas AJ, Burn DJ, Rowan EN, Littlewood E, Newby J, Cousins D, et al. A comparison of the efficacy of donepezil in Parkinson's disease with dementia and dementia with Lewy bodies. Int J Geriatr Psychiatry. 2005;20(10):938-44.

8. Molloy SA, Rowan EN, O'Brien JT, McKeith IG, Wesnes K, Burn DJ. Effect of levodopa on cognitive function in Parkinson's disease with and without dementia and dementia with Lewy bodies. J Neurol Neurosurg Psychiatry. 2006;77(12):1323-8.

9. Mori S, Mori E, Iseki E, Kosaka K. Efficacy and safety of donepezil in patients with dementia with Lewy bodies: preliminary findings from an open-label study. Psychiatry Clin Neurosci. 2006;60(2):190-5.

10. Rowan E, McKeith IG, Saxby BK, O'Brien JT, Burn D, Mosimann U, et al. Effects of donepezil on central processing speed and attentional measures in Parkinson's disease with dementia and dementia with Lewy bodies. Dement Geriatr Cogn Disord. 2007;23(3):161-7.

11. Edwards K, Royall D, Hershey L, Lichter D, Hake A, Farlow M, et al. Efficacy and safety of galantamine in patients with dementia with Lewy bodies: a 24-week open-label study. Dement Geriatr Cogn Disord. 2007;23(6):401-5.

12. Kurlan R, Cummings J, Raman R, Thal L. Quetiapine for agitation or psychosis in patients with dementia and parkinsonism. Neurology. 2007;68(17):1356-63.

13. Goldman JG, Goetz CG, Brandabur M, Sanfilippo M, Stebbins GT. Effects of dopaminergic medications on psychosis and motor function in dementia with Lewy bodies. Mov Disord. 2008;23(15):2248-50.

14. Aarsland D, Ballard C, Walker Z, Bostrom F, Alves G, Kossakowski K, et al. Memantine in patients with Parkinson's disease dementia or dementia with Lewy bodies: a double-blind, placebo-controlled, multicentre trial. Lancet Neurol. 2009;8(7):613-8.

15. Levin OS, Batukaeva LA, Smolentseva IG, Amosova NA. Efficacy and safety of memantine in Lewy body dementia. Neurosci Behav Physiol. 2009;39(6):597-604.

16. Molloy S, Minett T, O'Brien JT, McKeith IG, Burn DJ. Levodopa use and sleep in patients with dementia with Lewy bodies. Mov Disord. 2009;24(4):609-12.

17. Emre M, Tsolaki M, Bonuccelli U, Destée A, Tolosa E, Kutzelnigg A, et al. Memantine for patients with Parkinson's disease dementia or dementia with Lewy bodies: a randomised, double-blind, placebo-controlled trial. Lancet Neurol. 2010;9(10):969-77.

18. Lucetti C, Logi C, Del Dotto P, Berti C, Ceravolo R, Baldacci F, et al. Levodopa response in dementia with lewy bodies: a 1-year follow-up study. Parkinsonism Relat Disord. 2010;16(8):522-6.

19. Mori E, Ikeda M, Kosaka K. Donepezil for dementia with Lewy bodies: a randomized, placebo-controlled trial. Ann Neurol. 2012;72(1):41-52.

20. Ikeda M, Mori E, Matsuo K, Nakagawa M, Kosaka K. Donepezil for dementia with Lewy bodies: a randomized, placebo-controlled, confirmatory phase III trial. Alzheimers Res Ther. 2015;7(1):4.

21. McKeith I, Aarsland D, Friedhoff L, Lombardo I, France N, Dworak H, et al. HEADWAY-DLB: A multinational study evaluating the safety and efficacy of intepirdine (rvt-101) in dementia with lewy bodies. Alzheimer's & Dementia. 2017;13:P936.

22. Elder GJ, Colloby SJ, Firbank MJ, McKeith IG, Taylor JP. Consecutive sessions of transcranial direct current stimulation do not remediate visual hallucinations in Lewy body dementia: a randomised controlled trial. Alzheimers Res Ther. 2019;11(1):9.

23. Murata M, Odawara T, Hasegawa K, Kajiwara R, Takeuchi H, Tagawa M, et al. Effect of zonisamide on parkinsonism in patients with dementia with Lewy bodies: A phase 3 randomized clinical trial. Parkinsonism Relat Disord. 2020;76:91-7.

24. Gratwicke J, Zrinzo L, Kahan J, Peters A, Brechany U, McNichol A, et al. Bilateral nucleus basalis of Meynert deep brain stimulation for dementia with Lewy bodies: A randomised clinical trial. Brain Stimul. 2020;13(4):1031-9.

25. Manabe Y. A Preliminary Trial in the Efficacy of Yokukansankachimpihange on REM Sleep Behavior Disorder in Dementia With Lewy Bodies. Front Nutr. 2020;7:119.

26. Maltête D, Wallon D, Bourilhon J, Lefaucheur R, Danaila T, Thobois S, et al. Nucleus Basalis of Meynert Stimulation for Lewy Body Dementia: A Phase I Randomized Clinical Trial. Neurology. 2021;96(5):e684-e97.
