# Supplementary material for: Clinical outcome measures in dementia with Lewy bodies trials: critique and recommendations
Source: Transl Neurodegener. 2022 May 2;11:24. doi: 10.1186/s40035-022-00299-w (PMC9059356; doi:10.1186/s40035-022-00299-w)
Supplement: Supplementary file 4 — Additional file 4. Table S4: Selected motor outcomes. [file 40035_2022_299_MOESM4_ESM.docx]

**Supplemental Table 4. Selected REM-sleep behavior disorder outcomes**

| Outcome | Rater | Detection | Reliability | Responsiveness | MCID | Used in trials |
| --- | --- | --- | --- | --- | --- | --- |
| RBDSQ | Patient | + | NE | NE | NE | No |
| RBDQ-HK | Patient | + | + | + | NE | No |
| Mayo Sleep Questionnaire | Informant | +* | + | NE | NE | No |
| RBD Severity Scale | Clinician - vPSG | +* | + | + | NE | Yes[1] |

+, good/adequate; +/-, acceptable performance is questionable/mediocre. * Evaluated in DLB population. MCID: minimal clinically important difference; NE: not evaluated; vPSG: video polysomnography. RBD = REM Sleep Behavior Disorder; RBDSQ = REM Sleep Behavior Disorder Screening Questionnaire; RBDQ-HK = REM Sleep Behavior Disorder Questionnaire Hong Kong.

**REFERENCES**

1. Stefani A, Santamaria J, Iranzo A, Hackner H, Schenck CH, Högl B. Nelotanserin as symptomatic treatment for rapid eye movement sleep behavior disorder: a double-blind randomized study using video analysis in patients with dementia with Lewy bodies or Parkinson's disease dementia. Sleep Med. 2021;81:180-7.
